# Computational identification of significant immunogenic epitopes of the putative outer membrane proteins from *Mycobacterium tuberculosis*

**DOI:** 10.1186/s43141-021-00148-9

**Published:** 2021-03-29

**Authors:** Shobana Sundar, Lokesh Thangamani, Shanmughavel Piramanayagam

**Affiliations:** Computational Biology Lab, Department of Bioinformatics, Bharathiar University, Coimbatore, India

**Keywords:** Immunoinformatics, Outer membrane proteins, *Mycobacterium tuberculosis*, Epitopes

## Abstract

Novel vaccines are required to effectively combat the epidemic spread of tuberculosis. Using in silico approaches, this study focuses on prediction of potential B cell and T cell binding immunogenic epitopes for 30 putative outer membrane proteins of Mtb. Among these, certain immunodominant epitopes of Rv0172, Rv0295c, Rv1006, Rv2264c, and Rv2525c were found, which are capable of binding B-cell and a maximum number of MHC alleles. The selected immunodominant epitopes were screened for their allergenic and antigenic properties, their percentage identity against the human proteome and their structural properties. Further, the binding efficacy of the immunodominant epitopes of Rv0295c and Rv1006 with HLA-DRB1*04:01 was analyzed using molecular docking and molecular dynamics studies. Hence, the in silico-derived immunogenic peptides (epitopes) could potentially be used for the design of subunit vaccines against tuberculosis.

**Supplementary Information:**

The online version contains supplementary material available at 10.1186/s43141-021-00148-9.

## Introduction

Tuberculosis (TB) is caused by pathogenic bacillus *Mycobacterium tuberculosis* (Mtb) and is a deadly disease that affects millions of people worldwide. In accordance with the WHO Global tuberculosis report 2018, TB is one of the top ten causes for human deaths and estimated around 1.3 million deaths in HIV-negative people. Moreover, 10.0 million people developed TB disease in 2017. The emergence of multi-drug and extensively drug-resistant strains of Mtb increases the burden of the drug treatment regimen for TB. Currently, Bacille-Calmette-Guerin (BCG) is the only available vaccine for treating TB. In infants, it is shown to have a protective effect against tuberculous meningitis and miliary tuberculosis [28]. However, in adults, it is shown to have only limited protection against pulmonary TB. Moreover, it causes more severe complications such as suppurative lymphadenitis, osteomyelitis/osteitis, and disseminated BCG infection. Disseminated BCG infection is a severe adverse reaction that arises in people with impaired immunity. Therefore, the BCG vaccine is not being given for HIV positive patients and for infants born to HIV-positive mothers. Due to the limitations of the BCG vaccine, we need novel and effective vaccines against all forms of TB.

Immunoinformatics involves the use of computational tools to predict the immunogenic epitopes or peptides which could be used to design ideal subunit vaccine candidates. These tools simply use the organism’s genetic information, and it reduces the cost and time taken for the development of vaccines [8]. Subunit vaccines usually consist of certain immunoactive biomolecules such as polypeptides and glycolipids and usually, they need the help of an adjuvant for inducing immune protection. These can be easily prepared at low cost and highly specific and efficient with minimal side effects [18]. Many new and promising subunit TB vaccine candidates are in various stages of clinical trials [10, 12].

Outer membrane proteins (OMPs) play an important role in the host-pathogen interactions and in maintaining the integrity and permeability of the cell membranes. Due to their localization on the mycobacterial surfaces, they can be easily targeted by the host immune system and hence they are ideal candidates for vaccine design [14]. Recently, Baliga et al., in [5], have predicted immunogenic epitopes of the OMPs of the pathogen *Vibrio anguillarum*. Similarly, Rauta et al., in [22], have predicted immunogenic epitopes of the OMP's of the pathogen *Vibrio cholerae*. Song et al., in [27], have identified 144 putative OMPs of Mtb which could play some crucial role in mycobacterial pathogenesis. In this study, using computational approaches we intend to identify the potential immunogenic epitopes of 30 putative OMPs of Mtb. We believe that this study will provide suitable leads for the design of peptide-based subunit vaccines using OMP’s of Mtb.

## Methods

**Fig. 1:**
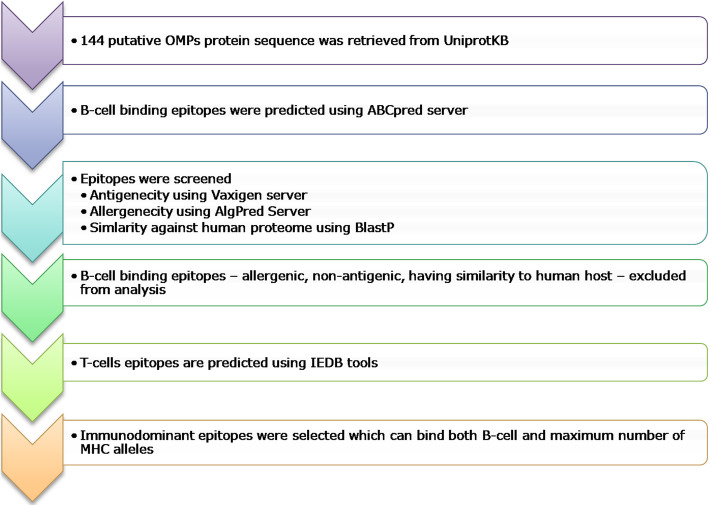
The overall methodology adopted in this study

The overall methodology adopted in this study to determine potential vaccine candidates of the putative OMPs of Mtb is depicted in Fig. 1.

### Sequence retrieval

FASTA Sequences of 143 putative OMPs of Mtb were retrieved from UniProtKB protein database and subjected for epitope prediction. UniprotKB Ids of the retrieved sequence is given in Table S1. The protein sequence of Rv1784 (one of the putative OMP) was not found in UniprotKB database.

### Sequence-based B cell prediction

B cell epitope prediction for the retrieved FASTA sequences of the OMP’s of Mtb was performed using IEDB tools. BepiPred Linear Epitope prediction method [16] was employed which uses a propensity scale of amino acids and Hidden Markov models for the prediction of potential immunogenic B cell epitopes. Default parameters were employed for the prediction.

### Evaluation of antigenic and allergenic properties of the predicted epitopes

Antigenic and allergenic values of the predicted B cell epitopes were calculated using VaxiJen (antigenic proteins should possess a score above 0.4) [9] and AlgPred servers (non-allergenic protein sequences should possess a score lesser than − 0.4) [24] using default parameters.

### Homology of the epitopes with the human proteome

The B cell binding epitopes were further screened for their similarity against humans, in order to avoid cross-reactivity. BLASTp program [2] was used to check the similarity of the epitopes against humans. The default non redundant protein sequences (nr) database was employed for similarity searching. All the other parameters were set to default values. The epitopes having lesser than 80% similar to the human proteome were further analyzed for its structural properties.

### T cell epitope prediction

The predicted B cell epitopes were further subjected for T cell binding prediction. The MHC-I binding predictions were made using the IEDB analysis resource Consensus tool [13] which combines predictions from ANN aka NetMHC (4.0) [3, 17, 20], SMM [21] and Comblib [26]. The reference set of 27 MHC-I alleles was used for the prediction [32]. The peptide length was set to 10. The high affinity binding epitopes were selected based on their percentile rank, which is set to 20.

The MHCII binding predictions were made using the IEDB analysis resource Consensus tool [30, 31]. The reference set of 27 MHC-II alleles was used for the prediction [11]. The peptide length was set to 15. The high affinity binding epitopes were selected based on their percentile rank, which is set to 20.

### Selection of immunodominant epitopes (IDEs) of the putative OMPs of Mtb

Immunodominant epitopes (IDEs) are regions which can bind B cell as well as maximum number of MHC-I and MHC-II alleles. The identification of IDEs has immense potential as it can lead to strong immune response and it can be effectively used to design peptide based vaccines. This method of finding IDEs was successfully employed by Verma et al. in [29] for the design of DnaK peptide vaccine against *S. typhi*.

### Prediction of transmembrane topology and the solubility of the epitopes

Structural properties of the IDEs such as solvent accessibility, transmembrane topology, and solubility upon overexpression were predicted using ACCpro, ABTMpro, and SOLpro tools respectively, found in the SCRATCH protein prediction server [7]. Solvent accessibility of the epitopes is an important criterion as the epitopes should be exposed for the interaction of the immune cells. Prediction of transmembrane topology for the epitopes is important because proteins spanning the membrane are difficult to clone and express; therefore, epitopes which are non-transmembrane proteins could be ideal vaccine candidates. The predicted epitopes should also be soluble on over-expression, so the solubility check was also performed.

### Molecular docking and molecular dynamics studies of the IDEs with HLA-DRB1*04:01

The selected IDEs of Rv0295c and Rv1006 were modeled using PEPFOLD 3 server [25] and were made to dock with HLA-DRB1*04:01(PDB ID: 5JLZ) using Cluspro server [15]. The higher ranked epitope-HLA complex was further subjected to molecular dynamics studies using GROMACS 2019 [1] software for 20ns. For the MD setup, GROMOS 43a1 force field was used and the epitope-HLA complex was placed in a cubic box filled with spc water molecules. The complex was neutralized by adding corresponding ions and energy was minimized using steepest-descent algorithm. Further, the complex was subjected to NVT and NPT equilibration steps for 100 ps, each. The temperature and the pressure were fixed at 300 K and 1 bar, respectively. Finally, the all atom MD run was performed for 20ns. The coordinates was written for every 10 ps. RMSD and RMSF of the epitope-HLA complex was computed using the GROMACS in-built tool namely, rms. Xmgrace was used to plot the graph.

## Results

Song et al., in [27], have identified 144 putative OMPs of Mtb and we have used this list of OMPs for the prediction of potentially immunogenic epitopes. B cell epitopes have been predicted for all the 144 OMP’s of Mtb and its antigenic and allergenic properties have been calculated. The B cell epitopes which are allergenic, non-antigenic are not considered for further analysis. Moreover, in order to avoid cross-reactivity, the predicted B cell epitopes whose similarity is greater than 80% against the human proteome were further excluded from our analysis. By applying all the above criteria’s, we have predicted B cell binding epitopes for 30 putative OMPs of Mtb. Additionally, to predict IDEs for the putative OMP’s of Mtb, the B cell binding epitopes were further subjected to T cell binding prediction.

The list of B cell epitopes predicted from the 30 putative OMPs of Mtb, along with their Vaxijen and AlgPred scores and the number of MHC alleles capable of binding these epitopes is given in Table 1. Further, we selected certain IDEs (given in Table 1) which are predicted to bind B-cell and the maximum number of MHC alleles (at least capable of binding > 25 alleles each from Class I as well as Class II). Further, the selected IDEs were checked for solvent accessibility, transmembrane topology, and solubility upon overexpression.

**Table 1:** List of potential immunogenic epitopes of the putative OMPs of Mtb

S. No	Putative OMPs of Mtb	B cell epitopes			T cell prediction		VaxiJen Score	AlgPred Score	Percentage identity to human proteome
		Start	End	Sequence	MHC Class I	MHC Class II			
1.	Rv0172^a^	382	432	ASTASTLPKEIAYSEPRLQPPNGYKDTTVPGIWVPDTPLSHRNTQPGWVVA	27	27	0.5031	− 0.79612523	No significant similarity found
2	Rv0257	21	52	GLRGSLPGDSGGTAPDSHRLPASSSPDGKNIG	19	0	0.778	− 0.49379	No significant similarity found
3	Rv0295c^a^	228	264	AIGQDPKLAPAPMLERQANQRSDEWVDRYRAEAPRLG	26	27	0.8884	− 1.24481	No significant similarity found
4	Rv0506	125	137	VKDERSETSPRAL	6	2	1.3782	− 0.7865	61.11%
5	Rv1006^a^	24	59	LNGCSSSASHRGPLNAMGSPAIPSTAQEIPNPLRGQ	26	27	0.4214	− 0.76213	No significant similarity found
6	Rv1351	1	34	MTPRSLPRYGNSSRRKSFPMHRPSNVATATRKKS	24	23	0.6134	− 0.79832	28.12%
7	Rv1477	238	269	SSEGGQGAPPFRMWDPGSGPAGGRAWDGLWDP	22	12	1.189	− 0.54733094	52.63%
8	Rv1478	211	230	MLEASGSAGKVTVSPVRKAG	18	10	0.9246	− 0.72966	57.89%
9	Rv1488	312	343	GKPGEDGVFRFEPSPVEDQPKHAADGDDAEVA	21	27	1.1083	− 0.42041	41.67%
10.	Rv1906c	110	135	CQPWQNTGSEGAAPAGVPGPEAGAQL	18	17	0.8377	− 0.4423	68.42%
11	Rv1910c	28	60	YGGNGDSRKAAPLAPKAAALGRSMPETPTGDVL	22	22	0.726	− 1.35881	42.86%
12	Rv2075c	392	403	SWAPDEPRAGAG	5	1	1.2283	− 0.62431	58.82%
13	Rv2112c	184	204	VTGSGRVGIGPSGDEPGFQLS	9	3	1.5556	− 0.41539	64.29%
14.	Rv2232	1	40	MSSPRERRPASQAPRLSRRPPAHQTSRSSPDTTAPTGSGL	24	5	0.7629	− 0.52367	No significant similarity found
		49	79	GIVTDTTASGTNCPPPPRAAARRASSPGESP	17	10	0.6672	− 0.73783	No significant similarity found
15.	Rv2264c^a^	383	425	AWSEADEDSHIGPAPGYTAARPSLSFDHDAHAEPEPKSPPIPW	27	27	0.7743	− 1.00823	52.17%
16	Rv2307c	115	135	GYGGNPGRPSEQGLAADARAA	12	2	0.9422	− 0.7492	64.71%
17	Rv2525c^a^	102	153	YGKGSTADWLGGASAGVQHARRGSELHAAAGGPTSAPIYASIDDNPSYEQYK	27	27	0.9026	− 0.48836	No significant similarity found
18	Rv2672	33	72	AFGADPRFATYSGAGPQGAATTTPPPAGPPPLAAPKNDLS	21	27	0.7615	− 0.65308	69.23%
19	Rv2891	33	52	PAHADDSRLGWPLRPPPAVV	21	4	1.2467	− 0.64531	75%
20.	Rv2956	199	247	AGALAGAGHRKSPKQGVFRGAAQGGDIVARQPPGRWVCPSSAGGPIGWH	22	25	0.4568	− 0.96573	36.59%
21.	Rv2980	33	55	NRQPPERPVVIPAVPAPQATGPG	16	27	0.5001	− 0.44976	68.75%
		68	93	GEYRRAPVAEPTTAGATAWRTGPNST	23	21	0.7059	− 0.75355	57.14%
22.	Rv3096	134	168	DPLPRPGRQRAPRAGVHNSGWVQSPGAERLDDRRY	24	11	0.4054	− 0.40957	No significant similarity found
23	Rv3212	1	13	MVKPERRTKTDIA	7	0	0.5383	− 0.47334	64.29%
24.		188	199	DARVKPSNRGLQ	4	2	1.3432	− 0.56986	66.67%
25.	Rv3484	358	380	AFGSAPPTSQTAAAAKPNPSTVV	17	13	0.6267	− 0.41964	62.5%
26.	Rv3492c	354	381	KTAQNDPSTVRGARNYPCQEFPGKRAPT	26	6	0.7796	− 0.48815	72.73%
		529	552	GAFADPAGGTGIFAPGMTGASSAE	21	15	0.5891	− 0.45661	71.43%
27.	Rv3587c	43	116	SSAGAKPVSADKPASAQSHPGSPAPQAPQPAGQTEGNAAAAPPQGQNPETPTPTAAVQPPPVLKEGDDCPDSTL	22	27	0.7745	− 0.76176	40.38%
28.	Rv3693	242	258	RVGVDPTAADPAGWPRL	17	0	0.5311	− 0.64804	75%
29.	Rv3796	139	182	LAPPGRAPVLVYGPGPAGGLPPSEVGNPNPATVNPANPTPGLAA	23	27	0.5826	− 0.45843	61.9%
30.	Rv3909	374	388	STRGATVLPDGPLTG	12	2	0.5078	− 0.43926	66.67%

^a^Selected IDEs

Five selected IDEs are discussed below:

1. ^382^ASTASTLPKE IAYSEPRLQPPNGYKDTTV PGIWVPDTPLSHRNTQPGWVVA^432^ of Rv0172 is predicted to be a B cell binding epitope and is predicted to bind all the 27 reference alleles of MHC Class I and Class II, respectively. Moreover, it is predicted to be antigenic and non-allergenic, cannot find significant similarity against the human proteome. This IE is a non-transmembrane protein; solvent exposed and is predicted to be soluble when over-expressed. Additionally, Rv0172 belongs to Mce (Mammalian cell entry) family of proteins which are crucial for the virulence of Mtb [34].
2. Similarly, we found a potential IE. ^228^AIGQDPKLAPA PMLERQANQRSDEW VDRYRAEAPRLG^264^B-cell binding epitope of Rv0295c is non-allergenic and antigenic and binds 26 alleles of MHC Class I and 27 alleles of MHC Class II. It has no sequence similarity with the human proteome, solvent-exposed, non-transmembrane protein and is predicted to be soluble when over-expressed. In fact, Rv0295c is a Trehalose 2-sulfotransferaseand it involves in catalyzing the transfer of a sulfuryl group from 3′-phosphoadenosine-5′-phosphosulfate (PAPS) to trehalose, which leads to the synthesis of trehalose-2-sulfate (T2S) [19].
3. The next IDE is “^24^LNGCSSSASHRG PLNAMGSPAI PSTAQEIPNPLRGQ^59^” from Rv1006 is predicted to be a B-cell binding epitope which also binds 26 and 27 alleles of MHC Class I and Class II alleles, respectively. It is antigenic, non-allergenic and has least similarity to the human host. Additionally, it is solvent exposed, non-transmembrane and soluble upon overexpression. Rv1006 is believed to be a conserved hypothetical protein.
4. “^383^AWSEADEDSHI GPAPGYTAARPSL SFDHDAHAEPEPKSPPIPW^425^” is predicted to be a B cell binding epitope from Rv2264c, it also binds all of the 27 reference alleles of Class I and Class II, respectively. It is also predicted to be antigenic, non-allergenic and has least similarity to the human proteome. It is solvent exposed non-transmembrane and soluble upon overexpression. Rv2264c is a conserved hypothetical protein.
5. “^102^YGKGSTADWLGGA SAGVQHARRGSELHA AAGGPTSAPIYA SIDDNPSYEQYK^153^” is predicted to be a B cell binding epitope from Rv2525c, it also binds all of the 27 reference alleles of Class I and Class II, respectively. It is also predicted to be antigenic, non-allergenic and has least similarity to the human proteome. It is solvent exposed non-transmembrane and soluble upon overexpression. Rv2525c is a tat secreted protein and it functions as a putative peptidoglycan hydrolase [6].

### Molecular docking and molecular dynamics studies

The epitopes of Rv0295c and Rv1006 was modeled using PEPFOLD 3 server and was subjected to molecular docking studies with the 3D structure of HLA-DRB1*04:01 using Cluspro server. The other epitopes could not be modeled by the PEPFOLD 3 server as the length of the epitope was greater than 50 amino acids. The epitope of Rv2264c was having 52.17% similarity (Table 1) with the human proteome was also excluded for molecular docking and dynamics studies. The top-ranked epitope-HLA-DRB1*04:01 complex was retrieved. The binding energies for the top ranked epitope-HLA-DRB1*04:01 complex is given in Table 2.

**Table 2:** Binding energy for the epitopes-HLA-DRB1*04:01 complexes

Gene name	Epitopes	Binding energy with HLA-DRB1*04:01 retrieved using Cluspro tool
Rv0295c	^228^AIGQDPKLAPAPMLERQANQRSDEWVDRYRAEAPRLG^264^	− 297.5
Rv1006	^24^LNGCSSSASHRGPLNAMGSPAIPSTAQEIPNPLRGQ^59^	− 270.4

**Fig. 2:**
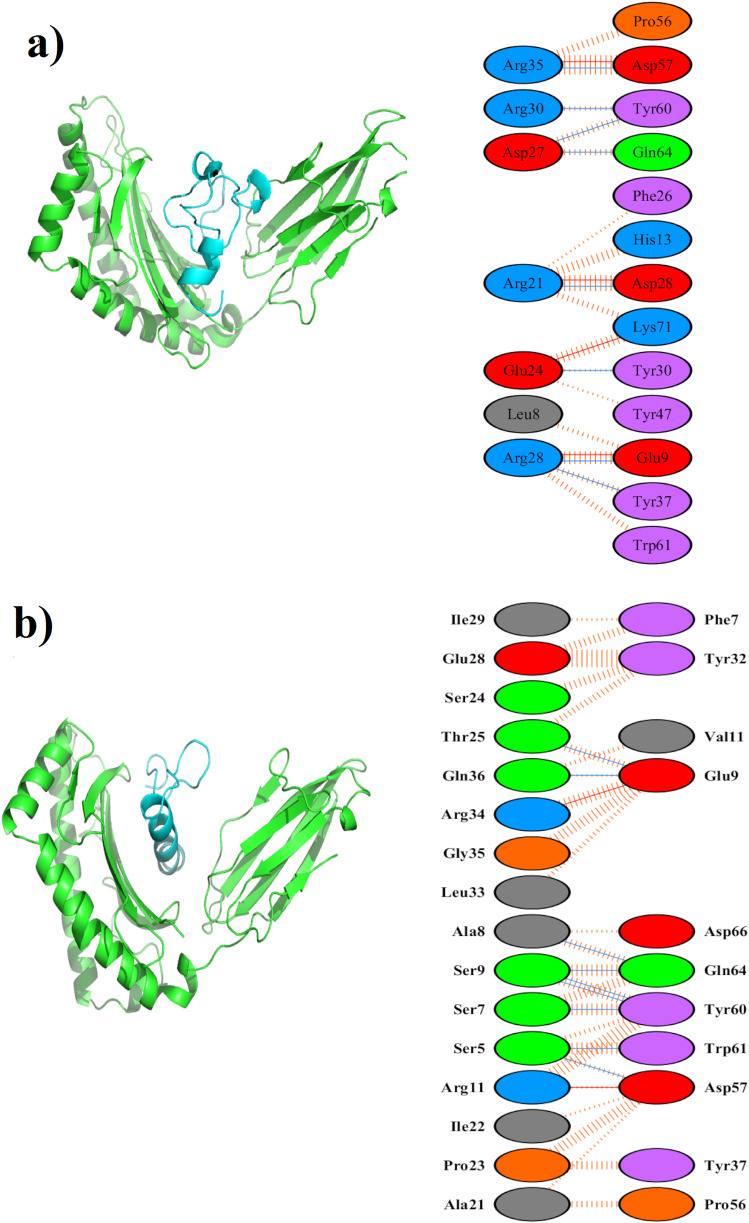
Molecular docking studies. **a** Docked complex of IDE of Rv0295c-HLA-DRB1*04:01. Green denotes HLA-DRB1*04:01 and Cyan denotes IDE of Rv0295c. Interactions of IDE of Rv0295c-HLA-DRB1*04:01 are shown. **b** Docked complex of Rv1006-HLA-DRB1*04:01. Green denotes HLA-DRB1*04:01 and Cyan denotes IDE of Rv1006. Interactions of IDE of Rv1006-HLA-DRB1*04:01. Interactions of IDE of Rv1006-HLA-DRB1*04:01 are shown

**Fig. 3:**
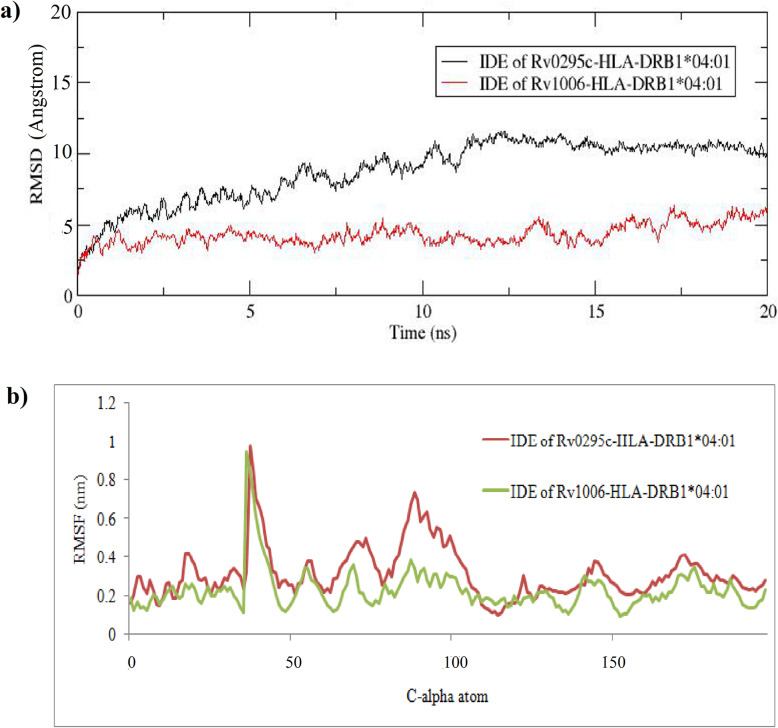
Molecular dynamics studies. **a** RMSD graph of HLA-DRB1*04:01 in Rv0295c-HLA-DRB1*04:01 and in Rv1006-HLA-DRB1*04:01 complexes. **b** RMSF plot of HLA-DRB1*04:01 in Rv0295c-HLA-DRB1*04:01 and in Rv1006-HLA-DRB1*04:01 complexes

The epitopes of Rv0295c and Rv1006 had strong affinity (Fig. 2a, b) with HLA-DRB1*04:01 and were further subjected to MD analysis. Each docked complex was subjected to a MD run for 20ns using GROMACS software. RMSD of HLA-DRB1*04:01 in Rv0295c-HLA-DRB1*04:01 complex (Fig. 3a) reached 10 Å around 10ns and remained the same until 20ns. Similarly, RMSD of HLA-DRB1*04:01 in the Rv1006-HLA-DRB1*04:01 complex reached 5 Å around 1ns and remained the same until 20 ns. The mobile regions (> 0.3 nm) of HLA-DRB1*04:01 depicted in Fig. 3b, mostly found to have interactions with the predicted epitope.

## Discussion

Vaccination is the best efficient method to treat TB. BCG is the currently available vaccine against TB. It expresses an Mtb immunodominant protein Antigen 85B (Ag85B) [23]. The antigen85 (Ag85) proteins comprise of Ag85A, Ag85B, and Ag85C. They are well-known mycolyltransferases or Diacylglycerolacyltransferases of Mtb which involves the transfer of mycolic acids to the cell wall arabinogalactan and they possess a high binding affinity for fibronectin [33]. BCG has been very effective against severe forms of TB in infants but it has protective efficacy against adults. Due to the adverse effects of BCG, a more effective and protective vaccine against all forms of TB is currently needed. In recent years, many new adjuvantedAg85B protein and vectored subunit vaccine candidates of Ag85A are in different phases of clinical trials. ESAT-6 and certain other proteins of Mtb have also been tested for their immunogenic competence [12].

Generally, bacterial OMPs serve as potential vaccine candidates, as their exposed epitopes on the bacterial cell surface could be easily recognized by the host immune system [5, 22]. In [35], Zvi et al. predicted 45 top-hits antigens covering the entire genome of Mtb as potential vaccine candidates which can be incorporated in the design of subunit vaccines against Mtb. Rv1813c, one of the 45 top-hits antigens predicted and is also predicted to be an OMP [27]. In 2015, Scientists at Infectious Disease Research Institute (IDRI) at Seattle, created a vaccine candidate ID93 which is a recombinant fusion of the four Mtb proteins. Notably, Rv1813c, a predicted OMP, is one of the four proteins of the ID93 vaccine candidate which has advanced to phase II clinical trials [4].

Therefore, from our in silico study of the OMP’s of Mtb, we have retrieved five IDEs (Table 1) which can bind both B cell and maximum number of T cells, antigenic, and non-allergenic, having lesser or no sequence similarity with the human proteome, non-transmembrane proteins and are predicted to be soluble when over-expressed. These five IDEs of the putative OMP’s (Rv0172, Rv0295c, Rv1006, Rv2264c, and Rv2525c) of Mtb could serve as ideal candidates for the design of subunit vaccines against tuberculosis.

## Conclusion

In this study, through immunoinformatics approach, potentially immunogenic epitopes for 30 putative OMPs of Mtb have been identified. Immuno dominant epitopes designed for Rv0172, Rv0295c, Rv1006, Rv2264c, and Rv2525c were predicted to be non-allergenic, antigenic and capable of binding B cells and a maximum number of MHC alleles. These epitopes also show lesser or no sequence similarity with the human proteome, solvent-exposed, non-transmembrane and soluble upon overexpression. Molecular docking and molecular dynamics analysis of Rv0295c and Rv1006 epitopes-HLA-DRB1*04:01 complex further enhance our study. Thus, we suggest that these in silico-derived epitopes could be useful in developing peptide-based subunit vaccines against tuberculosis.

## Supplementary Information

**Additional file 1: Table S1.** UniprotKB IDs of the putative OMPs of Mtb. **Table S2.** List of MHC-I and MHC-II alleles employed in the study. **Figure S3.** Ramachandran plot of IDE of Rv0295c. **Figure 4.** Ramachandran plot of IDE of Rv1006. **Figure S5.** Ramachandran plot of IDE of Rv2265.

## Data Availability

Not applicable.

## Abbreviations

TB: Tuberculosis
Mtb: *Mycobacterium tuberculosis*
HLA: Human leukocyte antigen
MHC: Major histocompatibility complex
IDE: Immunodominant epitope
WHO: World Health Organization
HIV: Human immunodeficiency virus
BCG: Bacille-Calmette-Guerin
OMP: Outer membrane protein

## References

[1] Abraham MJ, Murtola T, Schulz R (2015). GROMACS: High performance molecular simulations through multi-level parallelism from laptops to supercomputers. SoftwareX.

[2] Altschul SF, Gish W, Miller W, Myers EW, Lipman DJ (1990). Basic local alignment search tool. J Mol Biol.

[3] Andreatta M, Nielsen M (2016). Gapped sequence alignment using artificial neural networks: application to the MHC class I system. Bioinformatics.

[4] Baldwin SL, Reese VA, Po-wei DH (2016). Protection and long-lived immunity induced by the ID93/GLA-SE vaccine candidate against clinical Mycobacterium tuberculosis isolate. ClinVacc Immuno.

[5] Baliga P, Shekar M, Venugopal MN (2018). Potential outer membrane protein candidates for vaccine development against the pathogen Vibrio anguillarum: a reverse vaccinology based identification. Curr Microbiol.

[6] Bellinzoni M, Haouz A, Miras I, Magnet S, André-Leroux G, Mukherjee R, Shepard W, Cole ST, Alzari PM (2014). Structural studies suggest a peptidoglycan hydrolase function for the Mycobacterium tuberculosis Tat-secreted protein Rv2525c. J Struct Biol.

[7] Cheng J, Randall AZ, Sweredoski MJ, Baldi P (2005). SCRATCH: a protein structure and structural feature prediction server. Nucleic Acids Res.

[8] De Groot AS, Sbai H, Aubin CS (2002). Immuno-informatics: mining genomes for vaccine components. Immunol Cell Biol.

[9] Doytchinova IA, Flower DR (2007). VaxiJen: a server for prediction of protective antigens, tumour antigens and subunit vaccines. BMC Bioinformatics.

[10] Gong W, Liang Y, Wu X (2018). The current status, challenges, and future developments of new tuberculosis vaccines. Hum Vaccin Immunother.

[11] Greenbaum J, Sidney J, Chung J, Brander C, Peters B, Sette A (2011). Functional classification of class II human leukocyte antigen (HLA) molecules reveals seven different supertypes and a surprising degree of repertoire sharing across supertypes. Immunogenetics.

[12] Khoshnood S, Heidary M, Haeili M, Drancourt M, Darban-Sarokhalil D, Nasiri MJ, Lohrasbi V (2018). Novel vaccine candidates against Mycobacterium tuberculosis. Int J Biol Macromol.

[13] Kim Y, Ponomarenko J, Zhu Z, Tamang D, Wang P, Greenbaum J, Lundegaard C, Sette A, Lund O, Bourne PE, Nielsen M, Peters B (2012). Immune epitope database analysis resource.Nucleic. Acids Res.

[14] Koebnik R, Locher KP, Van Gelder P (2000). Structure and function of bacterial outer membrane proteins: barrels in a nutshell. Mol Micro bio.

[15] Kozakov D, Hall DR, Xia B, Porter KA, Padhorny D, Yueh C, Beglov D, Vajda S (2017). The ClusPro web server for protein–protein docking. Nat Protoc.

[16] Larsen JEP, Lund O, Nielsen M (2006). Improved method for predicting linear B-cell epitopes. Immunome Res.

[17] Lundegaard C, Lamberth K, Harndahl M (2008). NetMHC-3.0: accurate web accessible predictions of human, mouse and monkey MHC class I affinities for peptides of length 8–11. Nucleic Acids Res.

[18] Malonis RJ, Lai JR, Vergnolle O (2019). Peptide-based vaccines: current progress and future challenges. Chem Rev.

[19] Mougous JD, Petzold CJ, Senaratne RH, Lee DH, Akey DL, Lin FL, Munchel SE, Pratt MR, Riley LW, Leary JA, Berger JM, Bertozzi CR (2004). Identification, function and structure of the mycobacterial sulfotransferase that initiates sulfolipid-1 biosynthesis. Nat Struct Mol Biol.

[20] Nielsen M, Lundegaard C, Worning P, Lauemøller SL, Lamberth K, Buus S, Brunak S, Lund O (2003). Reliable prediction of T-cell epitopes using neural networks with novel sequence representations. Protein Sci.

[21] Peters B, Sette A (2005). Generating quantitative models describing the sequence specificity of biological processes with the stabilized matrix method. BMC Bioinfo.

[22] Rauta PR, Ashe S, Nayak D, Nayak B (2016). In silico identification of outer membrane protein (Omp) and subunit vaccine design against pathogenic Vibrio cholerae. Comput Biol Chem.

[23] Rizzi C, Peiter AC, Oliveira TL, Seixas Neto ACP, Leal KS, Hartwig DD, Seixas FK, Borsuk S, Dellagostin OA, Universidade Federal de Pelotas, Brasil, Universidade Federal de Pelotas, Brasil (2017). Stable expression of Mycobacterium bovis antigen 85B in auxotrophic M. bovis bacillus Calmette-Guérin. Mem Inst Oswaldo Cruz.

[24] Saha S, Raghava GPS (2006). AlgPred: prediction of allergenic proteins and mapping of IgE epitopes. Nucleic Acids Res.

[25] Shen Y, Maupetit J, Derreumaux P, Tufféry P (2014). Improved PEP-FOLD approach for peptide and mini protein structure prediction. J Chem Comp.

[26] Sidney J, Assarsson E, Moore C, Ngo S, Pinilla C, Sette A, Peters B (2008). Quantitative peptide binding motifs for 19 human and mouse MHC class I molecules derived using positional scanning combinatorial peptide libraries. Immunome Res.

[27] Song H, Sandie R, Wang Y, Andrade-Navarro MA, Niederweis M (2008). Identification of outer membrane proteins of Mycobacterium tuberculosis. Tuberculosis.

[28] Trunz BB, Fine PEM, Dye C (2006). Effect of BCG vaccination on childhood tuberculous meningitis and miliary tuberculosis worldwide: a meta-analysis and assessment of cost-effectiveness. Lancet.

[29] Verma S, Sugadev R, Kumar A, Chandna S, Ganju L, Bansal A (2018). Multi-epitope DnaK peptide vaccine against S. Typhi: an in silico approach. Vaccine.

[30] Wang P, Sidney J, Dow C, Mothé B, Sette A, Peters B (2008). A systematic assessment of MHC class II peptide binding predictions and evaluation of a consensus approach. PLoS Comput Biol.

[31] Wang P, Sidney J, Kim Y, Sette A, Lund O, Nielsen M, Peters B (2010). Peptide binding predictions for HLA DR, DP and DQ molecules. BMC Bioinfo.

[32] Weiskopf D, Angelo MA, de Azeredo EL, Sidney J, Greenbaum JA, Fernando AN, Broadwater A, Kolla RV, de Silva AD, de Silva AM, Mattia KA, Doranz BJ, Grey HM, Shresta S, Peters B, Sette A (2013). Comprehensive analysis of dengue virus-specific responses supports an HLA-linked protective role for CD8+ T cells. Proc Natl Acad Sci.

[33] Wiker HG, Harboe M (1992). The antigen 85 complex: a major secretion product of Mycobacterium tuberculosis. Microbiol Mol Biol Rev.

[34] Zhang F, Xie JP (2011). Mammalian cell entry gene family of Mycobacterium tuberculosis. Mol Cell Biochem.

[35] Zvi A, Ariel N, Fulkerson J, Sadoff JC, Shafferman A (2008). Whole genome identification of Mycobacterium tuberculosis vaccine candidates by comprehensive data mining and bioinformaticanalyses. BMC Med Genomics.

