# Supplementary material for: Computational identification of significant immunogenic epitopes of the putative outer membrane proteins from Mycobacterium tuberculosis
Source: J Genet Eng Biotechnol. 2021 Mar 29;19:48. doi: 10.1186/s43141-021-00148-9 (PMC8006519; doi:10.1186/s43141-021-00148-9)

**Supplementary Information**

**Table S1: UniprotKB IDs of the putative OMPs of Mtb**

| **List of OMPs** | **UniprotKB ID** | **List of OMPs** | **UniprotKB ID** | **List of OMPs** | **UniprotKB ID** | **List of OMPs** | **UniprotKB ID** |
| --- | --- | --- | --- | --- | --- | --- | --- |
| Rv0088 | P9WM73 | Rv1067c | Q79FT3 | Rv1973 | P9WJ77 | Rv3333c | O53383 |
| Rv0116c | O53638 | Rv1081c | O53429 | Rv1974 | O53975 | Rv3351c | O50380 |
| Rv0152c | Q79G05 | Rv1087 | Q79FT0 | Rv1975 | O53976 | Rv3388 | Q6MWX5 |
| Rv0164 | L7N657 | Rv1091 | Q79FS5 | Rv2075c | P9WLL5 | Rv3484 | O06347 |
| Rv0169 | Q79FZ9 | Rv1135c | P9WI29 | Rv2112c | P9WNU9 | Rv3492c | I6YGA5 |
| Rv0170 | O07414 | Rv1174c | O50430 | Rv2223c | P9WHR5 | Rv3494c | I6YC95 |
| Rv0171 | O07415 | Rv1184c | O50440 | Rv2224c | P9WHR3 | Rv3496c | I6XHD6 |
| Rv0172 | O07416 | Rv1209 | O05310 | Rv2232 | P9WPI9 | Rv3497c | I6YGB1 |
| Rv0174 | L0T2W6 | Rv1268c | P9WM47 | Rv2251 | L0TBR2 | Rv3498c | I6X7G8 |
| Rv0225 | P96407 | Rv1325c | P9WIF7 | Rv2253 | O53527 | Rv3499c | I6YC99 |
| Rv0241c | O53664 | Rv1339 | P9WGC1 | Rv2264c | O53538 | Rv3533c | P9WHX7 |
| Rv0257 | L7N694 | Rv1351 | P9WM17 | Rv2307c | P9WLC7 | Rv3547 | P9WP15 |
| Rv0295c | O53699 | Rv1375 | P9WF27 | Rv2353c | Q79FF3 | Rv3558 | Q6MWW0 |
| Rv0309 | O07236 | Rv1382 | P71810 | Rv2356c | P9WHZ7 | Rv3572 | I6X7P2 |
| Rv0320 | O07246 | Rv1386 | P9WIH1 | Rv2376c | P9WIN7 | Rv3587c | O53572 |
| Rv0403c | P9WJT5 | Rv1419 | P9WLX9 | Rv2396 | Q79FE6 | Rv3627c | O06380 |
| Rv0451c | P9WJS9 | Rv1433 | O06825 | Rv2525c | I6XEI5 | Rv3683 | I6X827 |
| Rv0455c | O53740 | Rv1477 | O53168 | Rv2565 | P9WIY7 | Rv3693 | O69661 |
| Rv0506 | P9WJT3 | Rv1478 | P9WHU5 | Rv2597 | P9WL73 | Rv3705c | I6XI06 |
| Rv0584 | O86365 | Rv1488 | P9WPR9 | Rv2599 | P9WL69 | Rv3749c | L0TGF0 |
| Rv0589 | Q79FY7 | Rv1548c | P9WI21 | Rv2668 | P71965 | Rv3796 | P72062 |
| Rv0590 | O07788 | Rv1566c | O06624 | Rv2672 | P71969 | Rv3802c | O53581 |
| Rv0592 | I6WYT7 | Rv1669 | O86371 | Rv2741 | Q79FB3 | Rv3878 | P9WJC3 |
| Rv0594 | O07784 | Rv1698 | P9WJ83 | Rv2799 | I6XFB7 | Rv3908 | P9WIX7 |
| Rv0614 | O07763 | Rv1800 | P9WI11 | Rv2840c | I6XFF7 | Rv3909 | O05436 |
| Rv0677c | P9WJS7 | Rv1803c | Q79FJ9 | Rv2891 | P9WL33 | Rv3916c | O53594 |
| Rv0679c | I6WZ30 | Rv1804c | O53953 | Rv2956 | I6Y242 | Rv3705c | I6XI06 |
| Rv0755c | P9WI37 | Rv1813c | P9WLS1 | Rv2980 | P95115 | Rv3749c | L0TGF0 |
| Rv0774c | I6Y8R4 | Rv1815 | P9WLR9 | Rv3004 | P9WIR1 | Rv3796 | P72062 |
| Rv0799c | I6Y4U9 | Rv1890c | O07742 | Rv3033 | I6YAY5 |  |  |
| Rv0817c | I6WZH9 | Rv1906c | O07726 | Rv3036c | I6YF08 |  |  |
| Rv0875c | P9WKR7 | Rv1910c | P9WFN5 | Rv3096 | I6YB54 |  |  |
| Rv0878c | P9WI35 | Rv1914c | O07719 | Rv3159c | Q6MX04 |  |  |
| Rv0888 | P9WKQ1 | Rv1955 | P9WJA5 | Rv3196 | O53342 |  |  |
| Rv0906 | P9WKP3 | Rv1966 | L7N698 | Rv3209 | O05857 |  |  |
| Rv0980c | Q79FU0 | Rv1967 | O53968 | Rv3212 | O05854 |  |  |
| Rv0988 | O86370 | Rv1968 | O53969 | Rv3224A | Q6MWZ5 |  |  |
| Rv0999 | O05582 | Rv1969 | O53970 | Rv3224B | Q6MWZ4 |  |  |
| Rv1006 | O05592 | Rv1971 | O53972 | Rv3267 | P96872 |  |  |

**Table S2: List of MHC-I and MHC-II alleles employed in the study**

| **MHC-I alleles** | **MHC-II alleles** |
| --- | --- |
| HLA-A*01:01 | HLA-DRB1*01:01 |
| HLA-A*02:01 | HLA-DRB1*03:01 |
| HLA-A*02:03 | HLA-DRB1*04:01 |
| HLA-A*02:06 | HLA-DRB1*04:05 |
| HLA-A*03:01 | HLA-DRB1*07:01 |
| HLA-A*11:01 | HLA-DRB1*08:02 |
| HLA-A*23:01 | HLA-DRB1*09:01 |
| HLA-A*24:02 | HLA-DRB1*11:01 |
| HLA-A*26:01 | HLA-DRB1*12:01 |
| HLA-A*30:01 | HLA-DRB1*13:02 |
| HLA-A*30:02 | HLA-DRB1*15:01 |
| HLA-A*31:01 | HLA-DRB3*01:01 |
| HLA-A*32:01 | HLA-DRB3*02:02 |
| HLA-A*33:01 | HLA-DRB4*01:01 |
| HLA-A*68:01 | HLA-DRB5*01:01 |
| HLA-A*68:02 | HLA-DQA1*05:01/DQB1*02:01 |
| HLA-B*07:02 | HLA-DQA1*05:01/DQB1*03:01 |
| HLA-B*08:01 | HLA-DQA1*03:01/DQB1*03:02 |
| HLA-B*15:01 | HLA-DQA1*04:01/DQB1*04:02 |
| HLA-B*35:01 | HLA-DQA1*01:01/DQB1*05:01 |
| HLA-B*40:01 | HLA-DQA1*01:02/DQB1*06:02 |
| HLA-B*44:02 | HLA-DPA1*02:01/DPB1*01:01 |
| HLA-B*44:03 | HLA-DPA1*01:03/DPB1*02:01 |
| HLA-B*51:01 | HLA-DPA1*01:03/DPB1*04:01 |
| HLA-B*53:01 | HLA-DPA1*03:01/DPB1*04:02 |
| HLA-B*57:01 | HLA-DPA1*02:01/DPB1*05:01 |
| HLA-B*58:01 | HLA-DPA1*02:01/DPB1*14:01 |

Figure S3: Ramachandran plot of IDE of Rv0295c


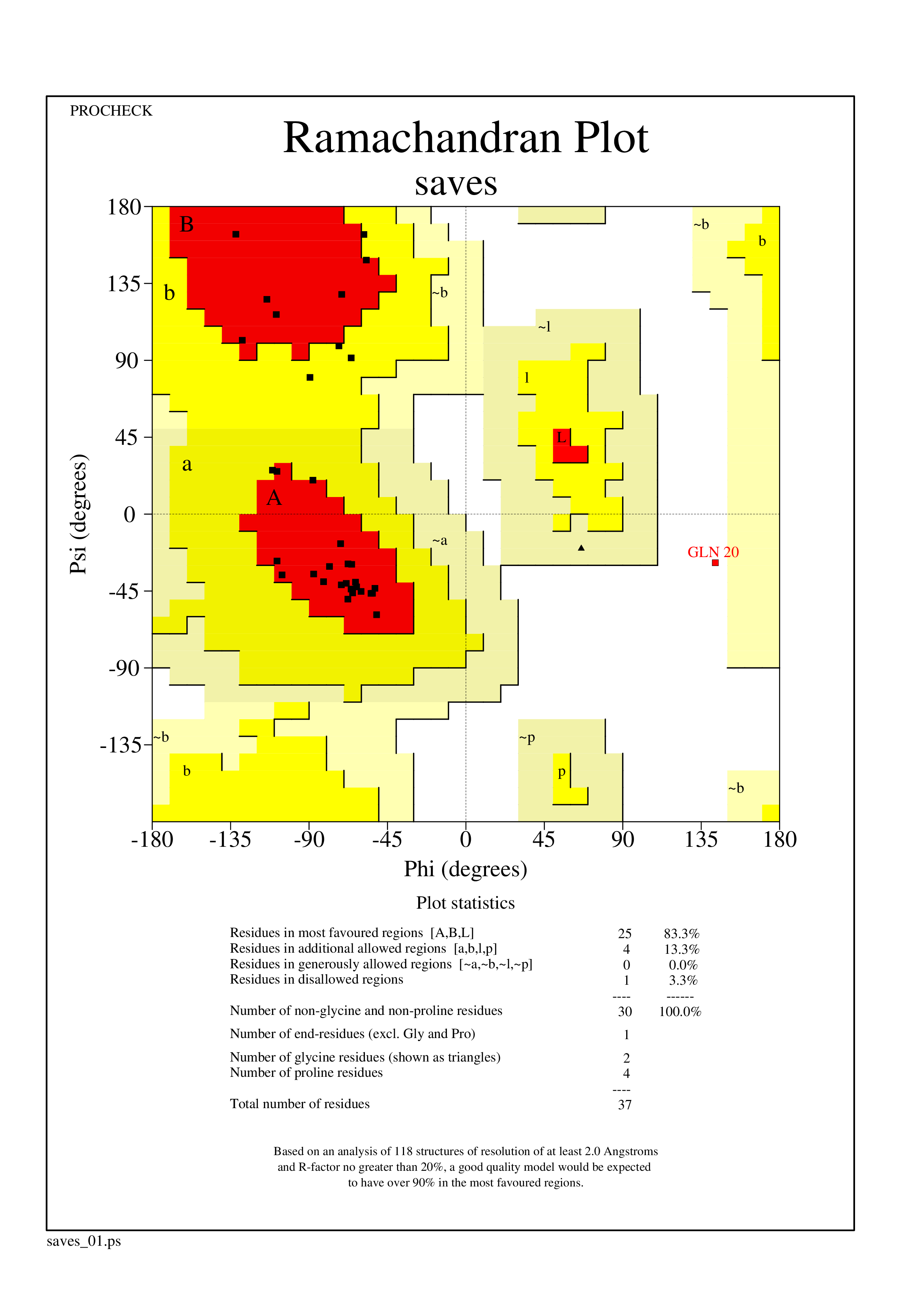


Figure S4: Ramachandran plot of IDE of Rv1006


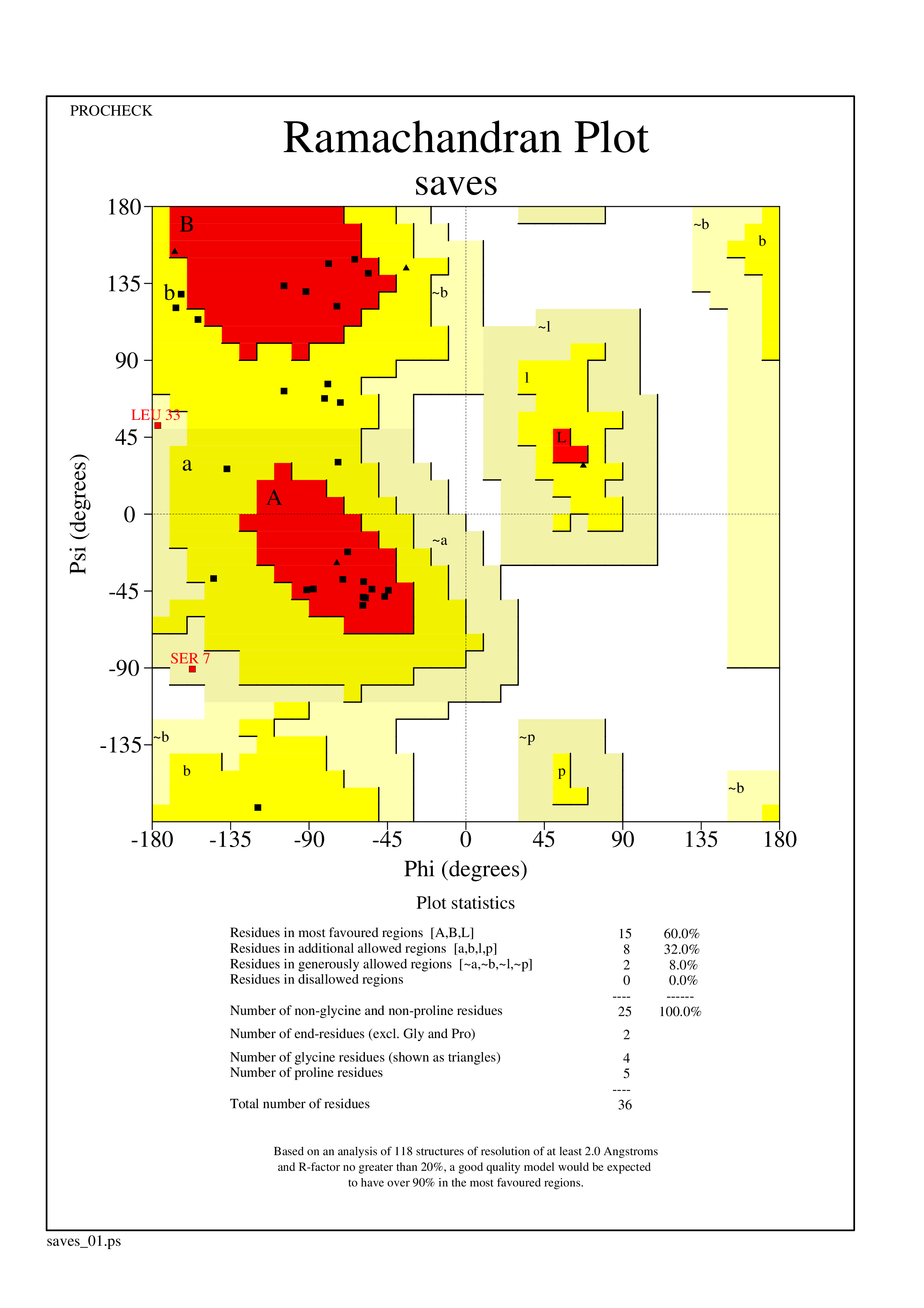


Figure S5: Ramachandran plot of IDE of Rv2265


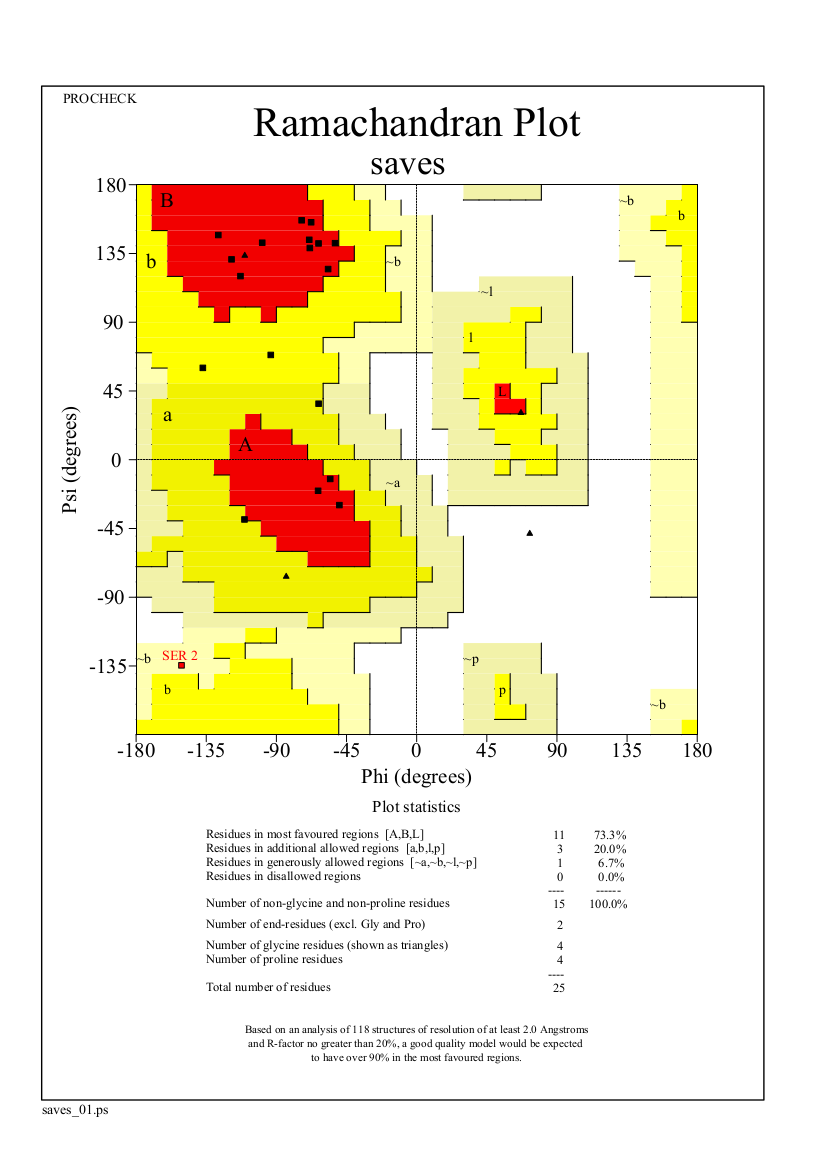

Supplement: Supplementary file 1 — Additional file 1: Table S1. UniprotKB IDs of the putative OMPs of Mtb. Table S2. List of MHC-I and MHC-II alleles employed in the study. Figure S3. Ramachandran plot of IDE of Rv0295c. Figure 4. Ramachandran plot of IDE of Rv1006. Figure S5. Ramachandran plot of IDE of Rv2265. [file 43141_2021_148_MOESM1_ESM.docx]
